# EMPOWERING OLDER ADULTS IN HEALTHCARE SETTINGS: COMMUNICATION SKILLS TRAINING FOR SURGICAL RESIDENTS

**DOI:** 10.1093/geroni/igz038.1094

**Published:** 2019-11-08

**Authors:** Linda Roberts, Charles Cornell, Mathias Bostrom, Sandra Goldsmith, Titilayo Ologhobo, Timothy Roberts, Laura Robbins

**Affiliations:** 1 Hospital for Special Surgery, New York, New York, United States; 2 NYU Langone Medical Center, New York, New York, United States

## Abstract

Older adults often perceive themselves as stigmatized and powerless in healthcare settings. Communication with them is complicated by age-related issues and negative stereotypes about older adults and aging. It is therefore vital for physicians and surgeons, who encounter the most vulnerable elderly, to communicate successfully with this population, who wish to maintain quality and dignity in their lives. Successful patient communication leads to better recall of information, compliance, adherence to medications, satisfaction, and overall better outcomes. We developed a two-part training program comprised of small group interactive didactic sessions on aging issues with third year surgical residents, and workshop demonstrations given by the residents to a group of older adults, followed by a question and answer session. Residents were assessed using a 22-item pre–post questionnaire covering medical knowledge of aging, attitudes toward older adults, and personal anxiety about aging. Since its inception, the program has reached 88 residents and 711 older adults. For residents, knowledge scores (p ≤ 0.001), six of nine attitude items (p ≤ 0.01) and one of four anxiety items (p ≤ 0.001) improved significantly. This is notable as well since attitudes and anxiety levels are attributes that are deep-seated and hard to change. For older adults, post surveys showed that 96% strongly agreed/agreed that residents had demonstrated sensitivity toward them and 96% were very satisfied/satisfied with the program. Our replicable, low-cost program enables residents to learn and realistically practice universal underlying communication skills in order to maintain effective and sensitive communication with this vulnerable population.

